# Typhoid Carriers

**Published:** 1912-06

**Authors:** 


### BUFFALO MEDICAL JOURNAL

**A Monthly Review of Medicine and Surgery**

*EDITOR*

A. L. BENEDICT.

All communications, whether of a literary or business nature, books for review and exchanges, should be addressed to the editor, 354 Franklin Street, Buffalo, N. Y Please make personal or telephonic calls before 1 p. m

The Buffalo Medical Journal will publish regularly. full transactions of any professional organization in western New York at cost. Personal notices, brief reports of society proceedings, and the like, are always welcome and will be published withou charge.

Subscriptions may commence at any time. $2.00 per year in advance,

Vol. LXVII. JUNE, 1912 No. 11

*There are no round trip tickets on the D. & O. road.*

### Typhoid Carriers

DR. WILBUR A. SAWYER (*Jour. A.M.A.,* May 4, 1912), reports 27 cases of typhoid with four deaths, due to a male carrier on Pacific ships. Edward R. Bigelow (idem) refers to a previously reported case, apparently male, from which 213 cases arose, and to several others, causing smaller epidemics, and suggests that the Widal test may be useful to detect them. Frederick M. Meader of Syracuse, at the latest meeting of the Medical Society of the State of New York, reported good results from immunization although, in the discussion, the point was raised whether immunization could be expected to prove efficient, on the ground that in the healthy carrier, the bacilli were probably merely saprophytes and not growing in or producing reactions with somatic structures. Abraham Jacobi stated that he had refused to take an active part in a crusade against carriers until water and milk supplies had been brought to a satisfactory condition.

All of these problems must be considered sub judice, requiring careful checking of theoretic with empiric knowledge. While the whole subject of "carriers" is of comparatively recent date and while the previous knowledge of diphtheria carriers did not, for a long time, suggest generalizations which now seem both obvious and practical, it may now be held that the medical profession is pretty well aroused to the importance of "carriers" in both an academic and a clinical and hygienic sense. At the same time, we ought to realize thoroughly that actual information as regards details, is extremely meagre and that every suspicious case should be fully investigated, both with reference to immediate prophylaxis and to more remote possibilities of developing general means of detection, immunization and disinfection.

The exact status of the parasite and host, in carriers, is a matter of the widest importance. For instance, if specific (active) immunization proves efficient in the treatment of typhoid carriers, we must conclude either that the carrier, though symptomatically healthy, is still in a state of disease, either locally or generally; or else that immunization produces an effect that is virtually extra-corporeal and which may indeed be considered as closely analogous to an excretion.

The extent to which germ-carrying applies to infections generally, is another problem of the highest interest, practically and theoretically. A clear-cut distinction between adventitious and essential conveyance of disease is already possible in many instances, if we regard such extremes as scarlet fever distributed by a furry pet which does not, itself ever contract the disease, so far as we know, and, on the other hand, malarial conveyance by anopheles. But closer study shows that there are many instances in which the distinction can not be made.

Another question of importance is whether "carrying," in this sense, is necessarily a sequel of an active infection. In the case of diphtheria, we know that persons in good general health may carry bacilli in the throat or even have a mild local reaction—the so-called follicular amygdalitis occurring in persons exposed to diphtheria and croup, noted by our predecessors. It is not impossible that many infections may exist in atypic, relative forms, not producing marked or even appreciable symptoms and still representing gtnuine reactions to the specific germ, and being contagious or infectious in more than an adventitious sense.

Some of the reports mentioned throw doubt on the common opinion that typhoid carriers are mainly females. A little reflection will convince us that females are more likely to arouse suspicion as carriers, indeed more likely to disseminate the disease on account of their more frequent employment in domestic service and of their coming more closely and more continuously in contact with the inhabitants of a given house or institution.

There is one favorable point in Sawyer's case that, while not at all technical or scientific, deserves emphasis. The carrier, on learning of his career, "showed deep concern and voluntarily entered the hospital."

In Buffalo, for several years, a disproportionate number, sometimes an actual majority of typhoid cases, are found in hospitals. What is being done to prevent further infection from these cases? Is the routine disinfection, not only of faeces but of other discharges, being properly carried out? We know academically, that typhoid bacilli occur in the urine in about 20 per cent. of all cases. We also know, academically, that bacilli persist in various localities for varying lengths of time, short of the relatively permanent persistence noted in "carriers." One more thing we know academically—how to detect the presence of the typhoid bacillus. How far are these academic principles being made practical. Are our typhoid patients released from quarantine only after the demonstrated absence of bacilli, as in the case of diphtheria? What is the answer to these same questions in other places? Why not answer some of them, right in our own territory in such a way as to establish a precedent for the rest of the country?

